# Role of Preservation Solution in Human Aneurysmatic Aorta Harvest and Transport: A Comparative Analysis of Different Solutions for Tissue Injury Protection

**DOI:** 10.21470/1678-9741-2023-0434

**Published:** 2024-10-14

**Authors:** Carlos Alexandre Curylofo Corsi, Maria Cecília Jordani, Jéssyca Michelon-Barbosa, Vinicius Flora Dugaich, Fabiola Mestriner, Cláudia Tarcila Gomes Sares, Rodolfo Borges dos Reis, Paulo Roberto Evora, Mauricio Serra Ribeiro, Christiane Becari

**Affiliations:** 1 Department of Surgery and Anatomy, Division of Vascular and Endovascular Surgery, Faculdade de Medicina de Ribeirão Preto, Universidade de São Paulo, Ribeirão Preto, São Paulo, Brazil; 2 Department of Surgery and Anatomy, Division of Urology, Faculdade de Medicina de Ribeirão Preto, Universidade de São Paulo, Ribeirão Preto, São Paulo, Brazil; 3 Department of Surgery and Anatomy, Division of Cardiac Surgery, Faculdade de Medicina de Ribeirão Preto, Universidade de São Paulo, Ribeirão Preto, São Paulo, Brazil; 4 Department of Biological Sciences, Faculdade de Odontologia de Bauru, Universidade de São Paulo, Bauru, São Paulo, Brazil

**Keywords:** Saline Solution, Tissue Preservation, Cell Culture Techniques, Smooth Muscle Myocytes, Abdominal Aortic Aneurysm, Histidine, Nitrates, Nitrites.

## Abstract

**Introduction:**

Human aortic tissues in vitro are tools to clarify the pathophysiological mechanisms of the cardiovascular system, cell culture, and transplants. Therefore, this study aims to analyze and compare the preservation of human aneurysmatic aortic tissues in three different solutions.

**Methods:**

Six human abdominal aortic aneurysms were obtained from patients after surgical ablation. The aorta samples were incubated in different solutions - 0.9% normal physiological saline solution, Ringer’s lactate solution, and histidine-tryptophan-ketoglutarate solution (Custodiol®). Segments were collected at 0, 6, 24, and 48 hours. Creatine kinase and nitrate/nitrite were quantified for each incubation time. The tissue’s alpha-smooth muscle actin was analyzed by immunofluorescence.

**Results:**

There was a significant increase in creatine kinase formation in the normal saline group at 0 and 48 hours and in the Ringer’s lactate group at 0 and 48 hours (P=0.018 and P=0.028). The lower levels of creatine kinase and nitrate/nitrite and the aortic tissues’ morphological integrity show that histidine-tryptophan-ketoglutarate has better tissue protection. These data suggest that histidine-tryptophan-ketoglutarate induces a protective effect on smooth muscle cells, with less tissue depletion in the aortic aneurysm.

**Conclusion:**

This study compared three preservation solutions with the potential for human abdominal aortic aneurysm tissue preservation. The histidine-tryptophan-ketoglutarate solution reduced tissue injury and improved tissue preservation in human abdominal aortic aneurysm tissue samples.

## Highlights

HTK (Custodiol®) solution shows better preservation of the human aneurysmatic aorta compared to 0.9% NS and RL solutions. HTK/Custodiol® is the aortic sample transport solution that favors cell viability for VSMC growth.

## INTRODUCTION

**Table:** 

Abbreviations, Acronyms & Symbols				
AAA	= Abdominal aortic aneurysm		NOs	= Nitric oxide synthase
ATP	= Adenosine triphosphate		NOx	= Nitrate/nitrite
BMI	= Body mass index		NS	= Normal saline
BSA	= Bovine serum albumin		OCT	= Optimal cutting temperature
CK	= Creatinine kinase		PBS	= Phosphate-buffered saline
DAPI	= 4’,6-diamidino-2-phenylindole		PBS-T	= PBS Tween®
HTK	= Histidine-tryptophan-ketoglutarate		RL	= Ringer’s lactate
IF	= Immunofluorescence		SEM	= Standard error of the mean
NO	= Nitric oxide		VSMCs	= Vascular smooth muscle cells

The development of human tissue bioengineering protocols contributes to understand cell morphology, physiology, and associated pathologies and to new studies for pharmacological treatments and interventions^[1^,^2]^. These tools' success depends on essential factors for their development, mainly using products to maintain the same vitality *in vitro* as *in vivo* cells/tissues^[3]^. Therefore, tissue preservation solutions in cold ischemia are important to preserve or repair tissue damage, consecutively avoiding edema, acidosis, free radical damage, and tissue depletion^[4^-^6]^.

Among the commercial solutions described in the literature and commonly used for the preservation and transportation of human tissues, some stand out, such as 0.9% normal physiological saline solution (normal saline [NS]), Ringer’s lactate (RL) solution, and histidine-tryptophan-ketoglutarate (HTK) solution/Custodiol®^[4^,^7]^. These are widely used mainly because they have lower viscosity and greater tissue permeability, thus developing protective factors against acute cell death processes. In addition, they have low costs and easy access for purchase in hospitals, among other factors^[3^,^8^,^9]^.

The choice of specific means of preservation for transport and pre-culture was mainly based on studies found in the current literature related to oxidative phosphorylation through parameters involved in respiration and mitochondrial permeability, in addition to determination of oxygenation capacity, morphological integrity, apoptosis, and presence of nitric oxide (NO) in tissues, using these solutions^[3^,^7^,^9]^. In this way, the choice of preservation medium for transportation can influence the results of primary cell cultures and even transplantation results^[6]^.

However, the importance of these solutions to obtain samples for *ex vivo* experiments and cell cultures is not completely shown, especially in aneurysmatic aorta. The evaluation and comparison of NS, RL, and HTK as preservation solutions are of great value and have yet to be analyzed in preserving human abdominal aortic aneurysm (AAA) tissue. Therefore, we sought to investigate which preservation solution is more beneficial, presenting fewer injuries and providing better preservation of aortic tissue to enhance *in vivo* experiments, such as cell and tissue culture, and transplantation experiments.

## METHODS

This project was approved by the Research Ethics Committee of the Hospital das Clínicas de Ribeirão Preto-Faculdade de Medicina de Ribeirão Preto-Universidade de São Paulo (CAAE number 82879518.6.0000.5440). The study was developed and carried out following the Code of Ethics of the World Medical Association (Declaration of Helsinki) regarding experiments involving human beings, having the informed consent form provided to the participants, informing them that the samples were obtained for experimentation. Participants’ privacy rights were preserved.

### Demographic and Clinical Data

Demographic and clinical data were obtained from the hospital’s digital database by analyzing medical records. The parameters analyzed were age, sex, race, smoking (current or past), hypertension, overweight (body mass index > 25 kg/m^2^), coronary artery disease, chronic obstructive pulmonary disease, myocardial infarction, peripheral artery disease, diabetes mellitus, congestive heart failure, arrhythmia, and stroke. The data was collected, organized, and analyzed in tables.

### Experimental Research Design

AAA fragments were collected from six patients undergoing AAA repair surgery. The aorta was removed after surgical ablation and washed with NS solution to be transported to the laboratory and kept at 4°C. In the laboratory, inside a laminar flow chamber and in a sterile procedure, the tissue was dissected with a scalpel blade following the sagittal/longitudinal direction of the aorta, dividing it into three uniform parts of 5 mm each. Finally, each fragment collected was incubated in three preservation solutions and kept at 4°C.

After incubating the tissues in their respective solutions, segments were collected at time 0 (immediately at the time of immersion of the tissue in the solution), and after 6, 24, and 48 hours to analyze creatine kinase (CK), nitrate/nitrite (NOx), and immunofluorescence (IF). The preservative solutions tested, containing the active ingredient concentration, are described in Table 1.

**Table 1:** Description ‘of the preservative solutions tested in the experiments.

1	Solution Physiological	Composition (100 ml)	Acting profile
	Physiological solution 0.9%	9 mg/ml sodium chloride (NaCl) (sodium 154 mEq/L + chloride 154 mEq/L) + 1 ml water for injection (q.s.p)	Solution to replenish water and electrolytes and control metabolic alkalosis.
			Osmolarity: 308 mOsm/L
			pH: 4.5 - 7.0^[6]^
2	Ringer’s lactate solution	0.6 g NaCl + 0.03 g potassium chloride + 0.02 g calcium chloride + 0.32 g sodium lactate + water for injection (q.s.p)	Rehydration solution to replenish and restore hydroelectrolyte balance when there is a loss of fluids and chloride, sodium, potassium, and calcium ions. Prophylaxis and treatment of metabolic acidosis.
			Osmolarity: 274.4 mOsm/L
			pH: 6.0 - 7.5^[9]^
3	Histidine-tryptophan-ketoglutarate	15 mEq/L sodium + 10 mEq/L potassium + 0.015 mmol/Kg calcium	Intracellular cardioplegic solution for organ preservation, in the form of a crystalloid with a low concentration of sodium and calcium, acting to deplete sodium from the extracellular spaces and causing hyperpolarization of the plasma membrane of myocytes, thus providing protection against ischemia.
			Osmolarity: 285 - 315 mOsm/L
			pH: 7.02 - 7.20^[7]^

q.s.p.= a sufficient quantity for. Authors, adapted^[6^-^9]^

### Creatine kinase determination

CK analysis was performed to measure tissue damage, as described in the literature^[10^,^11]^. For the CK activity assay (CELM SB-190/CK-NAC kit), the ultraviolet kinetic assay method at 340 nm was used for *in vitro* diagnostic use, with the aid of the CELM SB-190 device with semi-automatic analyzer, using the CK-NAC kit (by LabTest - Thermo Scientific, Massachusetts, United States of America). The liquid reagent contained in the kit was diluted with 20 µl of the sample and allowed the preparation of the total volume to be analyzed. In the apparatus, CK catalyzes the phosphorylation of adenosine triphosphate (ATP), obtaining creatine and ATP. The catalytic concentration is determined by employing associated reactions of hexokinase and glucose-6-phosphate, based on the rate of formation of nicotinamide adenine dinucleotide phosphate, measured by an increment of absorbance at 340 nm, which is proportional to the amount of CK existing in the sample. The results obtained were expressed in U/L. In humans, reference values for serum or plasma (heparin or ethylenediaminetetraacetic acid) are 26 to 189 U/L for men and 26 to 155 U/L for women^[10^,^11]^.

### Nitrate and nitrite determination

The determination of NOx is an indirect analysis of NO formation, a gaseous and inorganic free radical present in intracellular and extracellular processes. NO plays a fundamental role in the vascular system, acting on tissue angiogenesis^[12^-^14]^. NOx can be metabolized *in vivo* to produce NO and other bioactive nitrogen oxides, serving as an alternative NO source to the classical L-arginine-NO-synthase pathway, particularly in hypoxic states^[15^,^16]^. Aortic tissue samples (5 µL) were injected into the reaction chamber containing the reducing agent (0.8% vanadium chloride in 1 N HCl) at 80°C, which converts NOx into NO in equimolar amounts^[14^-^16]^.

### Immunofluorescence

Characterization of alpha-actin in the segment of the human abdominal aorta obtained from patients with AAA (n=3) incubated in preservative solutions (NS, RL, and HTK) after 48 hours was done. IF assays were performed with dual alpha-actin staining and 4’,6-diamidino-2-phenylindole (DAPI) core staining to identify and evaluate the tissue’s morphological integrity^[17]^. After separating the tissues immersed in the solutions, the inclusion technique for frozen tissues with optimal cutting temperature (OCT) was used as follows: previously, the solvent OCT was added in the freezer at -20°C for three hours and then stored in a freezer at -80°C, until cutting. Histological sections were performed in cryocutting equipment (Leica CM 1850) at a temperature of -20°C, with a thickness of 6-10 µm.

Subsequently, the slides were washed with 3 ml of 60% acetone, leaving them to rest in the open air for 10 minutes, then they were washed twice with 3 ml of 10× Dulbecco’s phosphate-buffered saline (PBS) buffer solution (Sigma - Aldrich, Saint Louis, Missouri, United States of America), waiting for five minutes. After that, 3 ml of NH4Cl was added, letting it rest for five minutes, then washing it 5× with PBS again. Afterward, the slides were blocked with 4 ml of bovine serum albumin (BSA) (Sigma [#A7906-50G] - Aldrich, Saint Louis, Missouri, United States of America) at 10% and allowed to rest for 40 minutes. After blocking with 10% BSA, the slides were incubated with different primary antibodies and kept overnight at 4°C, namely: anti-alpha smooth muscle actin antibody [1A4] and anti-muse antibody (1:100, monoclonal, Abcam #7817), for alpha-actin labeling. On the following day, the slides were washed twice with 3 ml of PBS-Tween® (PBS-T) buffer solution (0.025%, pH 7.4) (Sigma - Aldrich, Saint Louis, Missouri, United States of America), reserving them for five minutes. After that, secondary antibodies (1:100) were added, waiting for one hour, as follows: Alexa Fluor 488 Polyclonal Antibody (anti-rabbit) (Invitrogen by ThermoFisher - Catalog # A-11094); following, washing was performed with PBS-T for five minutes, ending with a wash containing 3 ml of PBS, waiting for five minutes. Next, the nucleic stain was added with the DAPI dye (Sigma [#10236276001 - Aldrich, Saint Louis, Missouri, United States of America), with a dilution of 1 to 1000 µL, for five minutes, and then washed with distilled water three times, reserving for five minutes. Afterward, the slides were covered with coverslips, placed in Fluoromount-G™ Mounting Medium (Thermo Fisher Scientific [#00-4958-02] - Waltham, Massachusetts, United States of America), and ready for analysis. The slides prepared with IF were examined in an inverted microscope, model Axio Observer, with LSM 780 MP System (by Carl Zeiss, Jena, Germany). The image editions were performed using the Fiji software (ImageJ).

### Statistical Analysis

Data are presented as mean ± standard error of the mean. The data was assessed for normality using the Shapiro-Wilk test. CK and NOx analyses were done by unpaired Kruskal-Wallis tests (non-parametric). The alpha-actin was analyzed by ordinary one-way analysis of variance (parametric). The statistical significance was *P*<0.05. All statistical analyses were performed in GraphPad Prism 8.

## RESULTS

The demographic and clinical data are described in Table 2. A comparative analysis of the data shows that 100% of the participants were male and over 65 years of age with smoking history, which reflects current descriptions in the literature, where AAA affects more males^[2^,^8]^.

**Table 2:** Demographic and clinical characteristics of the patients selected for the experiments between the AAA and AAA-free groups.

Group	Number of subjects (%)
Sex	
Male	6 (100)
Female	0 (0)
Ethnicity	
White	5 (84.4)
Black	1 (16.6)
Yellow	0 (0)
Age (mean ± SEM)	72.6 ± 3.8
Smoking (current or past)	6 (100)
Comorbidities (%)	
Alcoholic	1 (16.6)
Hypertension	6 (100)
Obesity (BMI ≥ 25 kg/m^2^)	6 (100)
Coronary artery disease	0
Chronic obstructive pulmonary disease	0
Myocardial infarction	1 (16.6)
Peripheral arterial disease	0
Diabetes mellitus	1 (16.6)
Congestive heart failure	1 (16.6)
Arrhythmia	0
Cerebral vascular accident	0

AAA=abdominal aortic aneurysm; BMI=body mass index; SEM=standard error of the mean

Data are shown in absolute (n) and relative (%) frequencies

**Fig. 1:**
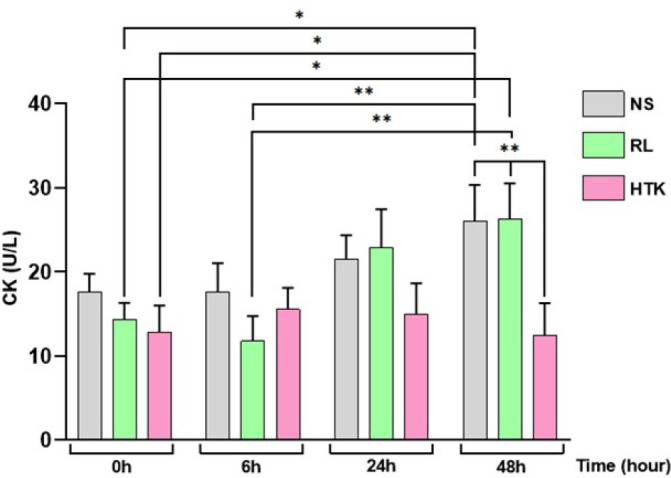
Creatine kinase (CK) determination in preservative solutions (normal saline [NS], Ringer’s lactate [RL], and histidine-tryptophan-ketoglutarate [HTK]) incubated with a fragment of human abdominal aorta obtained from patients with abdominal aortic aneurysm. CK quantification was performed at time 0 (0 hour), time 1 (6 hours), time 2 (24 hours), and time 3 (48 hours) (n=6). Values are expressed in µ/M. Data are presented as mean ± standard error of the mean, unpaired Mann-Whitney (non-parametric) test with a P<0.05% significance level (*P<0.05; **P<0.005).

CK was quantified to evaluate the degradation of the abdominal aortic tissue in NS, RL, and HTK at times 0, 6, 24, and 48 hours kept at 4°C. As shown in Figure 1 and Table 3, there was significance in the formation of CK in the NS group at times 0 and 48 hours (*P*=0.0183) and RL solution group at 0 and 48 hours (*P*=0.0287), and the HTK group showed no difference between the time 0 and after 48 hours. At 48 hours, there was a significant difference between the NS and HTK groups (*P*=0.037). HTK showed better protective effects for the aortic tissue, compared to RL and NS. These data suggest a protective effect of HTK on smooth muscle cell damage, with less free radical production and improved endothelial function.

**Table 3:** Creatine kinase (CK) and nitrate/nitrite (NOx) data in timeframes (0, 6, 24, and 48 hours).

	Timeframe											
	0 h			6 h			24 h			48 h		
	NS	RL	HTK	NS	RL	HTK	NS	RL	HTK	NS	RL	HTK
CK	17.5 ± 2.18	14.3 ± 1.93	12.7 ± 3.24	17.6 ± 3.40	11.8 ± 2.94	15.5 ± 2.56	21.4 ± 2.89	22.9 ± 4.55	14.9 ± 3.65	26.0 ± 4.29	26.3 ± 4.18	12.4 ± 3.86
NOx	2.15 ± 0.29	3.45 ± 0.46	2.20 ± 0.33	2.2 ± 0.25	4.61 ± 0.73	2.21 ± 0.18	2.56 ± 0.37	4.61 ± 0.71	2.28 ± 0.45	3.44 ± 0.34	5.65 ± 0.79	3.89 ± 0.23

HTK=histidine-tryptophan-ketoglutarate; NS=normal saline; RL=Ringer’s lactate

Data are presented as mean ± standard error of the mean

**Fig. 2:**
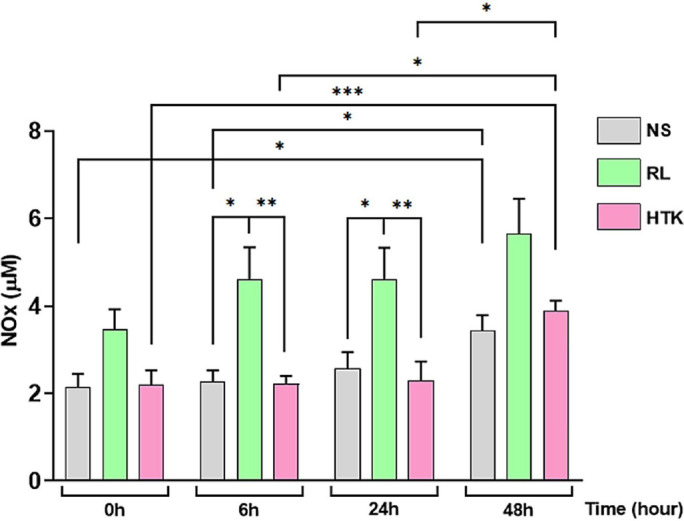
Determination of nitrate/nitrite (NOx) in preservative solutions (normal saline [NS], Ringer’s lactate [RL], and histidine-tryptophan-ketoglutarate [HTK]) incubated with a fragment of human abdominal aorta obtained from patients with abdominal aortic aneurysm. NOx quantification was performed at time 0 (0 hour), time 1 (6 hours), time 2 (24 hours), and time 3 (48 hours) (n=6). Values expressed in µ/M. Data are presented as mean ± standard error of the mean, unpaired Mann-Whitney (non-parametric) test with a P<0.05% significance level. (*P<0.05; **P<0.005; ***P<0.0005).

Figure 2 and Table 3 show NOx quantification, which was performed to evaluate the hypoxic status in the aortic tissue in NS, RL, and HTK solutions at times 0, 6, 24, and 48 hours and maintained at 4°C. At 6 hours, there was a significant difference between the NS group and the RL group (*P*=0.0221) and between RL solution and HTK (*P*=0.026). At 24 hours, there was a significant difference between the NS group and the RL group with *P*=0.0238. For RL and HTK groups, it was *P*=0.0172. In NS, there was a significant difference between times 0 and 48 hours (*P*=0.019), and times 6 and 48 hours (*P*=0.0242). Regarding the RL solution, there was no difference between the times verified. As for HTK, there was a significant difference between times 0 and 48 hours (*P*=0.0121), between times 6 and 48 hours (*P*=0.0095), and between 24 and 48 hours (*P*=0.0381).

**Fig. 3:**
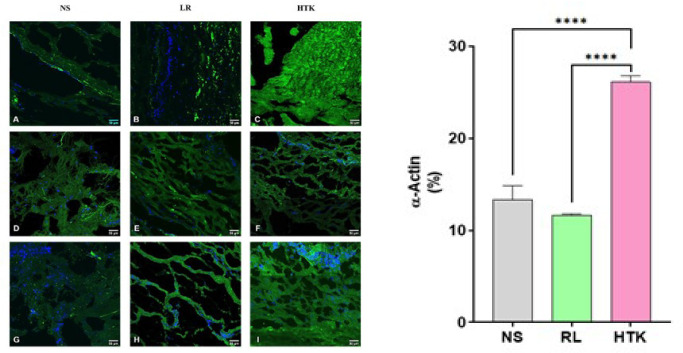
Photomicrograph of the characterization of alpha-actin in the segment of the human abdominal aorta obtained from patients with abdominal aortic aneurysm, incubated in preservative solutions (normal saline [NS], Ringer’s lactate [RL], and histidine-tryptophan-ketoglutarate [HTK]) after 48 hours. Immunofluorescence - A), B), C) patient 1; D), E), F) patient 2; G), H), I) patient 3 - evidencing the tunica media with smooth muscle fibers interspersed with elastic fibers with green coloring in the actin deposits, marked with anti-alpha smooth muscle actin antibody. Overlapping images with 4’,6-diamidino-2-phenylindole, in blue, show nucleated cells with important morphological differences. The cells were visualized in an inverted microscope (Axio Observer, LSM 780 MP). The overlapping of images (merge) was performed using the ImageJ - Fiji program (Bar = 50 µm, n=3 J). Quantification of histology media fluorescence intensity images obtained from the samples. Data showed the mean and standard error of the mean (n=3). The results were statistically analyzed using the t-test (parametric) with a P<0.05% significance level. (****P<0.00005)

As shown in Figure 3, among the three means of conservation for transport, HTK showed better protective effects for the alpha-actin integrity than RL and NS. There was a statistically significant difference in the preservation and fluorescence of smooth muscle actin fibers between NS and RL groups and the HTK group (*P*=0.0001). There was no statistical difference between NS and RL. IF assays were performed with double labeling for alpha-actin and DAPI staining of the nucleus to characterize and evaluate the tissue's morphological integrity.

A photomicrograph of the morphological characterization and development of subconfluent vascular smooth muscle cells, showing proliferative and multinucleated myoblasts, is provided in the Supplemental Digital Content (Link: https://youtu.be/SLq3eWaNhnM). The cells exhibit a typical spindle-shaped morphology with long projections connecting adjacent cells, indicating mitosis and phagocytosis.

**Supplemental Digital Content (SDC) 1:**
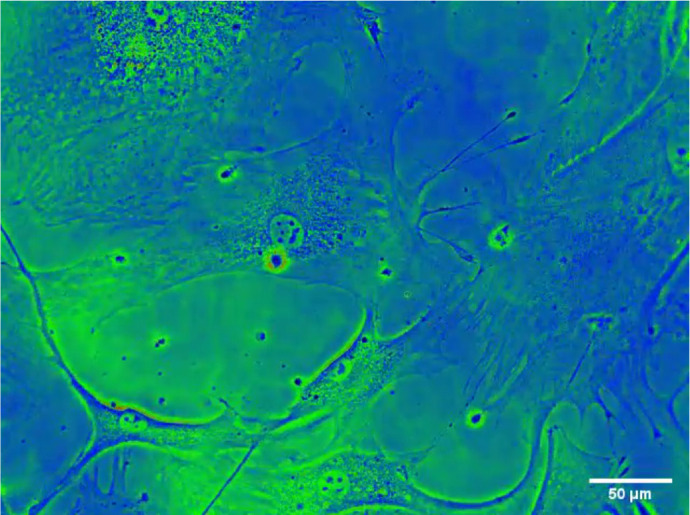
Photomicrograph of the morphological characterization and development of subconfluently vascular smooth muscle cells, showing proliferative and multinucleated myoblasts. It shows typical spindle-shaped morphology with long projections connecting adjacent cells, indicating mitosis and phagocytosis. Cells were visualized and filmed under an inverted microscope (Axio Observer, LSM 780 MP). The image superimposition (merge) was performed using the Image J - Fiji program. Bar = 50µm. (n = 1). Author’ name and videographer: Carlos Alexandre Curylofo Corsi. *Link: https://youtu.be/SLq3eWaNhnM*

## DISCUSSION

This study compared three preservation solutions with the potential for tissue preservation of human AAA, highlighting that the HTK solution improves tissue injury protection in human abdominal aortic aneurysm tissue preservation. There was an increase in CK formation in the NS and RL groups, but not in the HTK group. The lower levels of CK and NOx and the morphological integrity of the aortic tissues suggest that HTK has better tissue protection when compared to NS or RL. These data suggest that HTK induces a protective effect on smooth muscle cells and apoptosis, with less tissue depletion in the aortic aneurysm. The analysis of these solutions for preserving abdominal aneurysmatic aorta may improve vascular pathophysiological studies and vascular cell culture development.

Vivacqua et al.^[18]^ described the successful use of RL as an inhibitor of cellular apoptosis in fresh osteochondral connective tissue captured from patients, transporting these samples at 10°C. And yet, in association with antibiotics, they described that the solution could maintain tissue viability for allografts to be implanted in up to seven days. Our data demonstrate the lowest CK and NOx index in the tissue immersed in HTK, thus remaining in all comparison times, finally presenting the best results. Accompanied by RL, which presented the second-best results, and NS with the worst consequences.

A previous study using the human thoracic artery^[5]^ demonstrated a good performance of HTK in the tonus and relaxation of the vascular endothelium after 24 days in cold ischemia, however and in addition, our study demonstrated a better performance in tissue protection when comparing cell apoptosis markers. Using HTK for transporting and handling the samples allowed a significant advance in the standard operating procedure for primary cell culture, making it possible to isolate, purify, and grow smooth muscle cells from human aneurysmatic abdominal aortas. Our group has shown that maintaining aortic fragments on HTK improved the smooth muscle cells’ primary cell culture^[19]^.

The HTK, which has the essential amino acids in its composition as histidine, ketoglutarate, and tryptophan, showed the best results described here. The high concentrations of histidine found in this solution can justify its better performances because they are related to acidosis mitigants caused by the accumulation of anaerobic metabolites during cellular ischemia. With the presence of ketoglutarate, the production of ATP is also improved during the lack of perfusion, and tryptophan is also present in the solution, responsible for stabilizing the cell membrane of the tissue^[4^,^5^,^7]^.

Regarding muscle tissue degradation mechanisms, specifically smooth muscle cells, the activity of the isoenzyme CK stands out in the literature as a significant marker of tissue/cell injury^[20^,^21]^. Therefore, its specifications are intended for the determination of tissue degradation, mainly in the diagnosis of acute myocardial infarction and traumatic brain injuries, through the high values of CK-total^[10^,^11^,^20^-^23]^. Serum activities in blood plasma are rapidly increased from a stimulus of traumatic injury in some tissue and can be measured in the first two to six hours after muscle stretching, reaching their maximum values between 18 to 24 hours and may exceed 20 times their standard value, being one of the most sensitive tests for the diagnosis of coronary and cardiovascular diseases^[10]^. Furthermore, Thurner et al.^[23]^ identified and normalized free CK levels in cell cultures of smooth muscle from the intestine and rectum of rats to maintain cell vitality and protection. It is considered that the appearance of these isoenzymes reflects important changes that occur in the internal structures of the vessels and the musculature.

NO is generated endogenously from L-arginine through oxide synthase enzymes (nitric oxide synthase [NOs]). There are three different types of NOs, producers of NO, both encoded by different genes, which are: NOs1 (neuronal), NOs2 (inducible), and NOs3 (endothelial)^[24^-^26]^. Inside the cell, NO appears as a free radical and can quickly transform into nitrate (NO3-) or nitrite (NO2-), hydrophobically permeating the endothelium membranes of target tissues, thus resulting in a potent systemic vasodilator^[13^,^14]^. It is important to mention that, indirectly, high NO levels can lead to the production of reactive oxygen species, which are cytotoxic, pro-inflammatory, and even antimicrobial molecules. At low levels, they can lead to smooth muscle contraction, contributing to vascular pathologies^[12]^. The nitrate-nitrite-NO pathway, unlike the NOs-dependent pathway, is greatly enhanced during injury and hypoxia status^[27]^.

The alpha-actin measurement is a mark to evaluate the tissue’s morphological integrity. In the present study, the morphological integrity of the aortic tissues shows that HTK has better tissue protection when compared to the other two solutions. Smooth muscle cells show actin and myosin filaments responsible for the contraction and dilation of blood vessels, as well as the permeability of exchanges between the internal (interior of the vessel) and external environment, which results in the body’s homeostasis^[1^,^28]^.

There is an urgent need to develop new *in vitro* research protocols that mimic the pathophysiological environments of these diseases in human patients to study and treat them^[29^,^30]^. The choice of certain culture medium for transport and preservation was mainly based on the description of the benefits found and compared in the current literature, as well as the availability, easy access, and cost/benefit ratio of these products in loco in the surgical center and/or in the laboratory. It is proved that the development of the technique of transporting the aortic fragment in HTK, as well as the dissection and inversion of the vessels described, allowed the obtaining of viable vascular smooth muscle cells (VSMCs), being isolated and cultivated^[19]^. The study of aneurysmatic human aortic tissues *in vitro* is very important as a model to clarify the pathophysiological mechanisms of cardiovascular systems, cell culture, and transplants.

### Limitations

The limitation of this study is the small number of samples used as human aorta is not an easy sample to obtain. Also, another limitation was the fault of the human health aorta, as the control group, compared with the human AAA aorta.

## CONCLUSION

This study compared three preservation solutions with the potential for tissue preservation of human AAA, HTK solution reduced tissue injury or provided improved tissue preservation in human abdominal aorta aneurysm tissue samples.
